# RNA Nanovaccine Protects against White Spot Syndrome Virus in Shrimp

**DOI:** 10.3390/vaccines10091428

**Published:** 2022-08-30

**Authors:** Yashdeep Phanse, Supraja Puttamreddy, Duan Loy, Julia Vela Ramirez, Kathleen A. Ross, Ignacio Alvarez-Castro, Mark Mogler, Scott Broderick, Krishna Rajan, Balaji Narasimhan, Lyric C. Bartholomay

**Affiliations:** 1 Department of Entomology, Iowa State University, Ames, IA 50011, USA; 2 Pan Genome Systems, Madison, WI 53719, USA; 3 Merck Animal Health, Ames, IA 50010, USA; 4 Veterinary Diagnostics Center, University of Nebraska Lincoln, Lincoln, NE 68583, USA; 5 Department of Chemical and Biological Engineering, Nanovaccine Institute, Iowa State University, Ames, IA 50011, USA; 6 Instituto de Estadística, Universidad de la República, Montevideo 11200, Uruguay; 7 Department of Animal Science, Iowa State University, Ames, IA 50011, USA; 8 Department of Materials Science and Engineering, Iowa State University, Ames, IA 50011, USA; 9 Department of Materials Design and Innovation, University at Buffalo, Buffalo, NY 14260, USA; 10 Department of Pathobiological Sciences, University of Wisconsin-Madison, Madison, WI 53706, USA

**Keywords:** nanovaccine, dsRNA, polyanhydride, shrimp, WSSV

## Abstract

In the last 15 years, crustacean fisheries have experienced billions of dollars in economic losses, primarily due to viral diseases caused by such pathogens as white spot syndrome virus (WSSV) in the Pacific white shrimp *Litopenaeus vannamei* and Asian tiger shrimp *Penaeus monodon*. To date, no effective measures are available to prevent or control disease outbreaks in these animals, despite their economic importance. Recently, double-stranded RNA-based vaccines have been shown to provide specific and robust protection against WSSV infection in cultured shrimp. However, the limited stability of double-stranded RNA is the most significant hurdle for the field application of these vaccines with respect to delivery within an aquatic system. Polyanhydride nanoparticles have been successfully used for the encapsulation and release of vaccine antigens. We have developed a double-stranded RNA-based nanovaccine for use in shrimp disease control with emphasis on the Pacific white shrimp *L. vannamei*. Nanoparticles based on copolymers of sebacic acid, 1,6-bis(*p*-carboxyphenoxy)hexane, and 1,8-bis(*p*-carboxyphenoxy)-3,6-dioxaoctane exhibited excellent safety profiles, as measured by shrimp survival and histological evaluation. Furthermore, the nanoparticles localized to tissue target replication sites for WSSV and persisted through 28 days postadministration. Finally, the nanovaccine provided ~80% protection in a lethal WSSV challenge model. This study demonstrates the exciting potential of a safe, effective, and field-applicable RNA nanovaccine that can be rationally designed against infectious diseases affecting aquaculture.

## 1. Introduction

Shrimp aquaculture is an $18.3 billion industry providing employment to approximately 1 million people annually and has important implications for global food security [1,2]. The Pacific white shrimp, *Litopenaeus vannamei*, is the most frequently cultured species for human consumption [3]. Up to 40% of marine shrimp in production are lost annually due to infectious diseases of viral etiology, such as infectious myonecrosis virus (IMNV) and white spot syndrome virus (WSSV) [1]. In particular, WSSV is the most economically and globally significant shrimp pathogen decimating a tenth of shrimp production (~$1 billion) every year. Shrimp infected with WSSV develop white spots and succumb to the pathogen within 2–7 days, with mortality as high as 100% [4]. The lack of effective countermeasures against WSSV and other shrimp pathogens calls for urgent attention to developing disease intervention approaches that are safe and efficacious [5]. In this respect, vaccination strategies such as inactivated virus, subunit antigen, and DNA-based vaccines against WSSV have shown promise at the laboratory scale [6,7]. However, the drawbacks, such as variable efficacy, high manufacturing cost, and limited field applicability warrant further research [6].

RNA interference (RNAi) in invertebrates is an antiviral cellular mechanism by which a trigger, such as double-stranded RNA (dsRNA) or small interfering RNA (siRNA) starts sequence-specific degradation of target mRNA, thereby preventing viral gene expression. These dsRNA have been tested in a broad variety of settings, such as in plant pest control, next-generation mosquitocides, and vaccine design. In aquaculture systems, the concept of RNAi-based vaccines has been championed for several reasons: (a) RNAi works as an antiviral immune response in shrimp; (b) it is pathogen-specific; and (c) it generates a long-term protective immune response [5]. Previous work has shown that dsRNA-based vaccines are nontoxic, efficacious, and provide protection against a lethal dose of WSSV [8,9]. However, in all the applications mentioned above, the main challenge for the progression of a dsRNA-based vaccine from discovery to product and field application is its environmental stability and manufacturing costs. We posit that the limitations to dsRNA delivery in the field could be overcome using a nanocarrier delivery platform that further enhances cellular uptake, establishes long tissue residence times, is dose sparing, thereby reducing the cost and is adaptable for mass vaccinations. In this respect, biodegradable polyanhydride nanoparticles have proven to be efficacious delivery systems for antigen-based vaccines and drugs [10,11,12,13,14]. These carriers have been synthesized using copolymers, based on sebacic anhydride (SA), 1,6-bis(*p*-carboxyphenoxy)hexane (CPH) and 1,8-bis(*p*-carboxyphenoxy)-3,6-dioxaoctane (CPTEG), degrade via surface erosion [15]. The surface erosion ultimately excludes water from the bulk of the material and has been shown to provide enhanced stability of encapsulated payloads [16], which could be critical in an aquaculture setting. Furthermore, the ability to tailor copolymer compositions, and therefore the polymer hydrophobicity, has also been shown to enable sustained antigen release [16] as well as enhance antigen internalization by antigen-presenting cells [17,18].

In this proof-of-concept study, the first one to our knowledge, we combined two platform technologies—RNAi-based virus-specific dsRNA and a polyanhydride nanoparticle-based delivery platform—to develop a nanovaccine for aquaculture applications. The effect of nanoparticle chemistry on safety, biodistribution, and persistence was evaluated in vivo in *L. vannamei.* To mimic an oral route of administration, nanovaccines were injected via reverse gavage to target the gastrointestinal epithelia. Finally, nanovaccine efficacy was tested in a lethal WSSV challenge model in vivo in shrimp.

## 2. Materials and Methods

### 2.1. dsRNA Synthesis

For WSSV dsRNA generation, a plasmid with the potential to make a hairpin RNA was constructed for a WSSV gene with proven protective capacity [19]. This construct was transformed into an RNase III-deficient *E. coli* strain HT115 (DE3). Next, IPTG (isopropyl β-D-1-thiogalactopyranoside)-induced HT115 cells were pelleted and the WSSV 477 dsRNA was isolated using TRIzol reagent as per the manufacturer’s instructions (Thermo Fisher Scientific, Grand Island, NY, USA). The quantity and purity of the dsRNA was ascertained using NanoDrop ND-1000 (NanoDrop Technologies, Wilmington, NC, USA). Finally, the integrity of the dsRNA was checked using 1% standard agarose gel.

### 2.2. Polyanhydride and Nanoparticle Synthesis

Copolymers of 20:80 CPH:SA and 20:80 CPTEG:CPH were synthesized via melt polycondensation as previously described [20,21] and characterized using ^1^H nuclear magnetic resonance spectroscopy (VXR 300 MHz, Varian, Palo Alto, CA, USA) to assess composition, purity, and molecular weight. The number average molecular weights were approximately 20,154 Da and 8694 Da for 20:80 CPH:SA and 20:80 CPTEG:CPH, respectively, and consistent with previous work [20,21].

Polyanhydride nanoparticles were synthesized using a nanoprecipitation technique as previously described [22]. Briefly, 1 wt.% rhodamine B (Sigma Aldrich, St. Louis, MO, USA) and polymer were dissolved in methylene chloride at a concentration of 20 mg/mL. The solution was sonicated for 30 s before precipitating the nanoparticles into pentane (at a 1:250 ratio of methylene chloride:pentane). The nanoparticles were then collected and dried via vacuum filtration. During synthesis of 20:80 CPTEG:CPH nanoparticles, the pentane was chilled in a liquid nitrogen bath due to the lower glass transition temperature of CPTEG-containing polymers [21]. Nanoparticles encapsulating 11 wt.% dsRNA were synthesized following the same process. Particle morphology and size were characterized using scanning electron microscopy (FEI Quanta 250, FEI, Hillsboro, OR, USA) and found to be consistent with previous work [16,22].

### 2.3. Release Kinetics of dsRNA from Polyanhydride Nanoparticles

Nanoparticles loaded with 11 weight% dsRNA were incubated at a concentration of 10 mg nanoparticles per mL of saline (2% sodium chloride in RNase-free water) at 37 °C. Periodically, the nanoparticles were centrifuged to the bottom of the tube, and the saline containing released dsRNA was removed and replaced with fresh saline. The released dsRNA in the saline was quantified at each time point by reading the absorbance of the sample at 260 nm with a NanoDrop ND-1000 Spectrophotometer (NanoDrop Technologies) for approximately 1 month. To determine the encapsulation efficiency, the release of dsRNA was continued until the particles were completely degraded and no further dsRNA was detected within the saline. The encapsulation efficiency was then calculated by dividing the total amount of dsRNA released from the nanoparticles by the amount of dsRNA added during nanoparticle synthesis.

### 2.4. Stability of dsRNA Released from Polyanhydride Nanoparticles

The dsRNA-containing nanoparticles were sonicated in 2% artificial sea salt solution (1 mg particles/100 µL saline) and incubated for 1 hour at room temperature. After the incubation, the particles were centrifuged briefly and the supernatant containing released dsRNA was removed and analyzed on a standard 1% agarose gel.

### 2.5. Shrimp Rearing

Specific-pathogen free (SPF) *L. vannamei* were received from Shrimp Improvement Systems (Islamorada, FL, USA). Animals were maintained as previously described [23]. After arrival, animals were acclimatized for 1–2 weeks in 1 ton fiberglass tanks filled with artificial seawater made by mixing Crystal Sea Marinemix (Marine Enterprises International, Baltimore, MD, USA) with municipal water to a salinity of 28–30 parts per thousand (ppt). Water temperatures were maintained at 25–27 °C. Tanks were constantly aerated with air stone aeration and were outfitted with an oyster-shell airlift biofilter and activated carbon filter. Water quality, including ammonia and nitrite levels, was measured weekly. The animals were fed twice a day with Raceway plus commercial shrimp feed according to size and developmental stage (Zeigler Bros, Gardners, PA, USA).

### 2.6. Nanovaccine Safety

SPF shrimp (~3 g) were injected via reverse gavage with 500 µg of either 20:80 CPH:SA or 20:80 CPTEG:CPH nanoparticles suspended in 100 µL of water. Water-injected animals were used as controls. Survival was monitored until 60 days postinjection. Shrimp body weight was recorded at day 0 (initial weight) and at day 60 (final weight).

### 2.7. Histopathology

At the termination of the bioassays, live shrimp were fixed by injecting Davidson’s fixative (22% of 100% formalin, 33% of 95% ethanol, 11.5% glacial acetic acid and 33.5% distilled water) at multiple places in the cephalothorax and abdominal regions. Shrimp whole bodies were then immersed in fixative solution for 72 h at room temperature. Following fixation, shrimp were transferred to 75% ethanol. Shrimp were dissected into various tissue sections and a blinded histopathological analysis was performed at the Aquaculture Pathology Laboratory of the University of Arizona in Tucson, AZ, USA.

### 2.8. Biodistribution

SPF shrimp (~3 g) were injected via reverse gavage with 250 µg of 1% rhodamine-loaded 20:80 CPH:SA or 20:80 CPTEG:CPH nanoparticles in 100 µL water. Animals injected with water were used as controls. Animals were dissected into cephalothorax, hepatopancreas, gut, gills and stomach regions at 0, 2, 7, 14, 21 and 28 days postinjection. Fluorescent images of the dissected tissues were collected with an in vivo Multispectral FX Pro imaging system (Carestream, Rochester, NY, USA) using 510 nm excitation and 600 nm emission filter wavelengths. A white light image was also captured to define the tissue boundaries and regions of interest. Fluorescence measurements were performed at the same exposure setting to compare all data sets to one another. Image Analysis NIH Image Jv1.47 m (U.S. National Institutes of Health, Bethesda, MD, USA) was utilized to quantify the mean fluorescent intensities (MFI) values. Briefly, a region of interest (ROI) was drawn around each tissue sample utilizing the white light image. The same ROI was then applied to the fluorescent image and the MFI quantified utilizing the Analyze→Measure function [24,25]. The fold change in fluorescence was calculated by dividing the MFI of an individual tissue sample with the MFI of the corresponding tissue collected from controls. Experiments were performed in duplicate with 3–4 animals per group per time point in each experiment.

### 2.9. Virus Stock Preparation

Virus inoculum was prepared from tissue of WSSV-infected animals as described previously [23]. Tissue was collected from WSSV infected animals, macerated, and homogenized with sterile TN buffer (0.02 M Tris-HCl, 0.4 M NaCl, pH 7.4). The homogenate was clarified three times by centrifuging at 14,000× *g* for 30 min; 15,000× *g* for 15 min; and 25,000× *g* for 60 min. The final supernatant after clarification was diluted 1:10 (~6.83 × 10^6^ viral copy numbers/µL DNA) with 2% sodium chloride. The viral stock was then aliquoted and stored at −80 °C. For virus challenge experiments, the stock was diluted 1:7500 to get the final concentration of ~9.1 × 10^2^ viral copy numbers/µL DNA.

### 2.10. Bioassays for Survivability

SPF *L. vannamei* shrimp (~3 g) were divided into six experimental groups: (a) WSSV 477 dsRNA encapsulated in 20:80 CPTEG:CPH (Encap_477_ 20:80 CPTEG:CPH) nanoparticles; (b) WSSV 477 dsRNA encapsulated in 20:80 CPH:SA (Encap_477_ 20:80 CPH:SA) nanoparticles; (c) soluble WSSV 477 dsRNA (Soluble_477_); (d) soluble _eGFP_ dsRNA (Soluble _eGFP_) (non-shrimp sequence-specific vaccination control); (e) unvaccinated, unchallenged control (negative); and (f) sham-vaccinated (sham). Each experimental group contained a total of 30 shrimp (three replicates of 10 shrimp) except the negative control for which only 20 shrimp were used. Ten shrimp were stocked per fiberglass tank containing 50 gallons of artificial seawater and an oyster-shell airlift biofilter with 28–30 ppt salinity. A temperature of 28 °C was maintained throughout the course of experiment.

Following acclimatization for at least 3 days, 100 μL of the nanovaccine (100 μg of 11% dsRNA-loaded nanoparticles) was administered by reverse gavage to each shrimp to mimic the oral route of administration. The animals in the soluble dsRNA group were given 11 μg of dsRNA in water. The animals in the sham and negative control groups received water by reverse gavage. At 72 h postvaccination, all shrimp (except the negative control group) were challenged intramuscularly in the third abdominal segment with 100 µL of the WSSV virus stock (~ 9.1 × 10^2^ viral copy numbers/µL DNA). Shrimp were fed and monitored for mortality twice a day for 12–15 days postchallenge. Moribund shrimp were identified and fixed for histopathology. Dead shrimp were removed in a timely manner and stored at −80 °C to be tested for the presence of virus load by qPCR. At termination, two animals per group were fixed for histopathology and the remaining animals were stored at −80 °C to be tested for the presence of virus load by qPCR. These studies were performed in duplicate.

### 2.11. Quantitative Real Time PCR

DNA template from individual shrimp was extracted using the DNeasy blood and tissue kit (Qiagen, Valencia, CA, USA). Muscle tissue was homogenized using glass grinders followed by DNA extraction according to manufacturer’s instructions. The DNA was stored at −80 °C until use. Quantitative real-time PCR primers and standard were designed as described previously [26]. The primers used were WSSV forward primer: 5′-TGG TCC CGT CCT CAT CTC AG-3′; WSSV reverse primer: 5′-GCT GCC TTG CCG GAA ATT A-3′; and the probe: 5′-FAM-AGC CAT GAA GAA TGC CGT CTA TCA CAC A-TAMRA-3’. The qPCR reactions were performed using the Quantitect Probe kit (Qiagen) in 25 μL reactions containing 3 μL of DNA template, 12.5 μL of Master Mix, 0.5 μL of TaqMan probe, 1 μL of 20 μM WSSV forward primer, 1 μL of 20 μM WSSV reverse primer, and 7 μL of nuclease-free water. Each DNA sample was assayed in duplicate. Four different dilutions of standard were used to generate the standard curve. The reactions were run on a BioRad CFX96 real-time PCR machine (BioRad, Hercules, CA, USA) with cycling conditions including 15 min of preincubation at 95 °C followed by 35 cycles of 15 s at 95 °C and 1 min at 60 °C [16]. Virus copy number was calculated using CFX Manager software (BioRad) according to a standard curve.

### 2.12. Statistical Analysis

Statistical analysis was performed using JMP and SAS 9.4 software (SAS Institute, Cary, NC, USA). For comparisons of multiple treatment groups, data were analyzed using Tukey’s honestly significant difference (HSD). Differences were considered significant when *p* < 0.05. For viral challenge data, a binomial generalized linear model was fitted, with survival counts in the final day (12 DPI) as response variable and treatment levels and experiment as fixed effects. In order to improve parameter estimation, Bayesian models with normal vague priors for the regression coefficients were used. The models were fitted with proc Genmod. All models were run using 3 chains with 2000 iterations for burn-in and 10,000 for sampling after burn-in period, and convergence was studied with the Gelman–Rubin statistic.

### 2.13. Principal Component Analysis (PCA)

The ordering of axes, as defined by the eigenvalues corresponding with the loadings was used for defining parameters of multidimensional data, with the caveat that the first PC, PC1, captured a majority of the variance in the data [27,28]. The integration through PCA of discrete data with PCA parameterized continuous data was performed as described previously [29,30,31]. In all cases, the data were mean-centered prior to analysis, and when the units of the dimensions were different, then the data were normalized. The similarity of systems was defined through comparison of Euclidean distances, where a shorter Euclidean distance represents higher correlation or similarity.

## 3. Results

### 3.1. Polyanhydride Nanoparticles Provide Sustained Released of dsRNA

**Figure 1:**
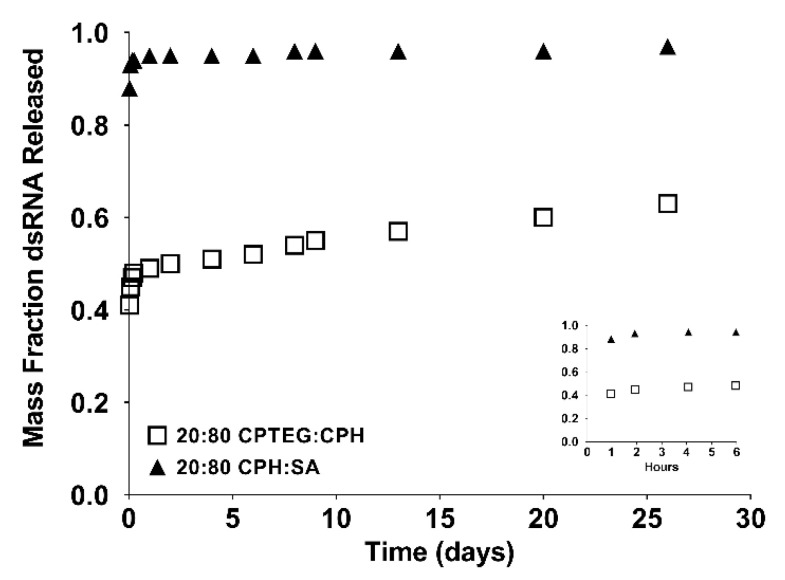
In vitro release kinetics of dsRNA-loaded nanoaparticles. Nanoparticles loaded with 11% WSSV 477 dsRNA were incubated in saline for approximately 1 month and the release kinetics were measured. Data are presented as the mean ± standard error of the mean (SEM) and are representative of 3 replicates per formulation.

Polyanhydride nanoparticles encapsulating 11% WSSV 477 dsRNA were successfully synthesized via nanoprecipitation. The average diameter of the resulting nanoparticles was determined using ImageJ (version 1.46 r, NIH, Bethesda, MD, USA) and found to be approximately 273 ± 126 nm (20:80 CPH:SA) and 219 ± 74 nm (20:80 CPTEG:CPH) for nanoparticles loaded with rhodamine (Table 1). Nanoparticles loaded with 11% dsRNA had a mean diameter of 290 ± 160 nm and 164 ± 66 nm for 20:80 CPH:SA and 20:80 CPTEG:CPH, respectively (Table 1) consistent with previous work [16,22]. The particles were then incubated in saline for approximately 1 month to observe the release kinetics of dsRNA. The release of dsRNA from both the 20:80 CPH:SA and 20:80 CPTEG:CPH nanoformulations displayed near-zero order release kinetics after an initial burst (i.e., within the first hour of release). It is known that the more hydrophobic CPH-rich copolymers have slower erosion rates [32,33]. Thus, the rate of dsRNA release from the 20:80 CPTEG:CPH nanoparticles was much slower than that from the 20:80 CPH:SA nanoparticles (Figure 1). In addition, it was observed that the initial burst and the dsRNA encapsulation efficiency were highly dependent on nanoparticle chemistry. Approximately 90% of the dsRNA encapsulated within the 20:80 CPH:SA nanoparticles was released within the first hour and the encapsulation efficiency of the dsRNA in these particles was 34%. By contrast, the dsRNA released from 20:80 CPTEG:CPH nanoparticles with a reduced initial burst (40%) and the encapsulation efficiency of the dsRNA in these particles was 74% (Figure 1 and Table 1).

### 3.2. dsRNA Released from Polyanhydride Nanovaccines Is Stable

**Figure 2:**
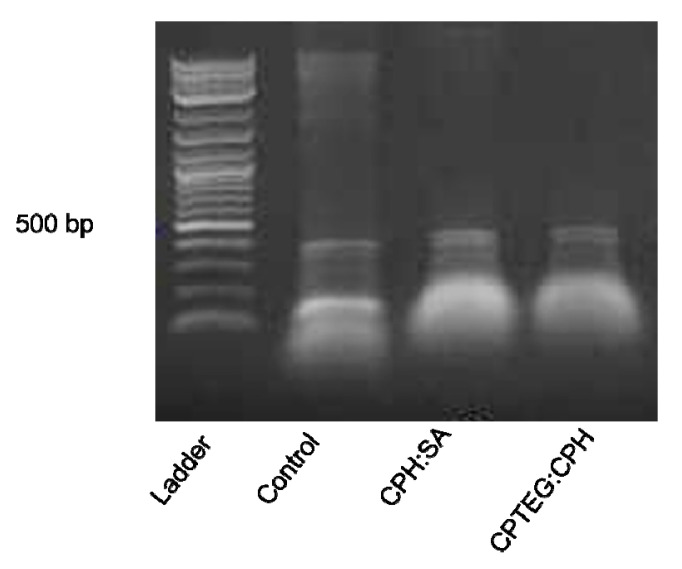
Stability of released dsRNA from nanoparticles. Released dsRNA from nanoparticles was evaluated for using gel electrophoresis.

To determine whether the encapsulation process had any detrimental effect on the stability of the dsRNA, an early time point postencapsulation was utilized. The dsRNA released from the 20:80 CPH:SA or 20:80 CPTEG:CPH nanoparticles was stable and no indication of degradation was observed in comparison to the unencapsulated dsRNA control (Figure 2). These results indicate the ability of the polyanhydride nanoparticles to release stable dsRNA payloads.

### 3.3. Polyanhydride Nanovaccines Do Not Induce Adverse Effects In Vivo

**Figure 3:**
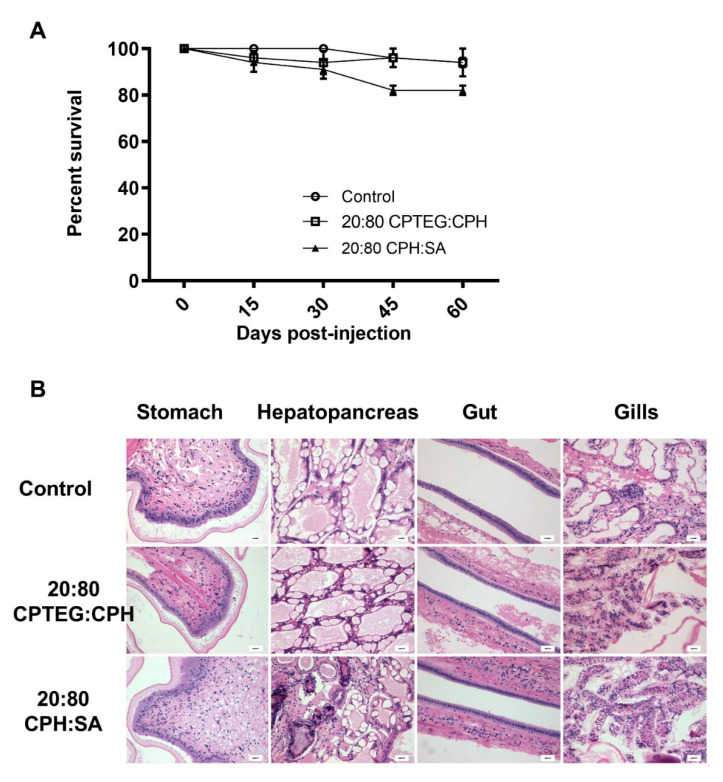
Safety profile of nanoparticles in shrimp. Shrimp were injected with 500 mg of either 20:80 CPTEG:CPH or 20:80 CPH:SA nanoparticles by reverse gavage (RG). (**A**) Shrimp survival was monitored through 60 days postinjection. Data are expressed as the mean ± SEM of three independent experiments. No statistical difference between groups at day 60. (**B**) Blinded histopathological analysis of tissue was performed by fixing live shrimp in Davidson’s fixative. Scale bar = 20 mm.

To test the biocompatibility of the nanoparticles, shrimp were subjected to a high dose (500 µg; 5-fold higher compared to the dose used for dsRNA delivery) of blank (i.e., empty) 20:80 CPH:SA or 20:80 CPTEG:CPH nanoparticles and monitored for survival, weight gain and histopathological changes. No significant differences in survival were observed 60 days postinjection between animals injected with water (95% survival) and animals injected with either 20:80 CPH:SA (85% survival) or 20:80 CPTEG:CPH (95% survival) nanoparticles (Figure 3A), affirming the excellent biocompatibility of these nanoparticles.

Because the goal of the vaccination regimen is to sustain shrimp aquaculture in a safe and efficient manner, changes in shrimp weight gain pre- and post-nanoparticle administration were investigated. Interestingly, the animals injected with 20:80 CPH:SA (10.17 g) or 20:80 CPTEG:CPH (10.52 g) nanoparticles had higher final weights than control animals (9.10 g) (Table 2). Most importantly, no weight loss was observed in the animals that received the nanoparticle formulations compared to control animals.

Finally, to ascertain that the nanoparticles did not induce any adverse effects in shrimp, multiple tissue types, including stomach, hepatopancreas, gut, and gills, were evaluated for histopathology in a blinded fashion. No gross histopathological lesions were observed in shrimp tissue injected with any of the nanoparticles compared to control animal tissues (Figure 3B). An in-depth analysis was performed to observe lymphoid organ spheroids, hemocytic nodules, hemocytic congestion (inflammation), and skeletal muscle necrosis because these are hallmark histopathological indicators of pathology caused by infection and stress in shrimp. The results demonstrated that animals injected with the 20:80 CPH:SA and 20:80 CPTEG:CPH nanoparticles exhibited excellent in vivo safety profiles and none of the markers were found to be significantly different compared to the control animals (Table 3).

### 3.4. Polyanhydride Nanoparticles Exhibit Chemistry-Dependent Spatiotemporal Distribution In Vivo

**Figure 4:**
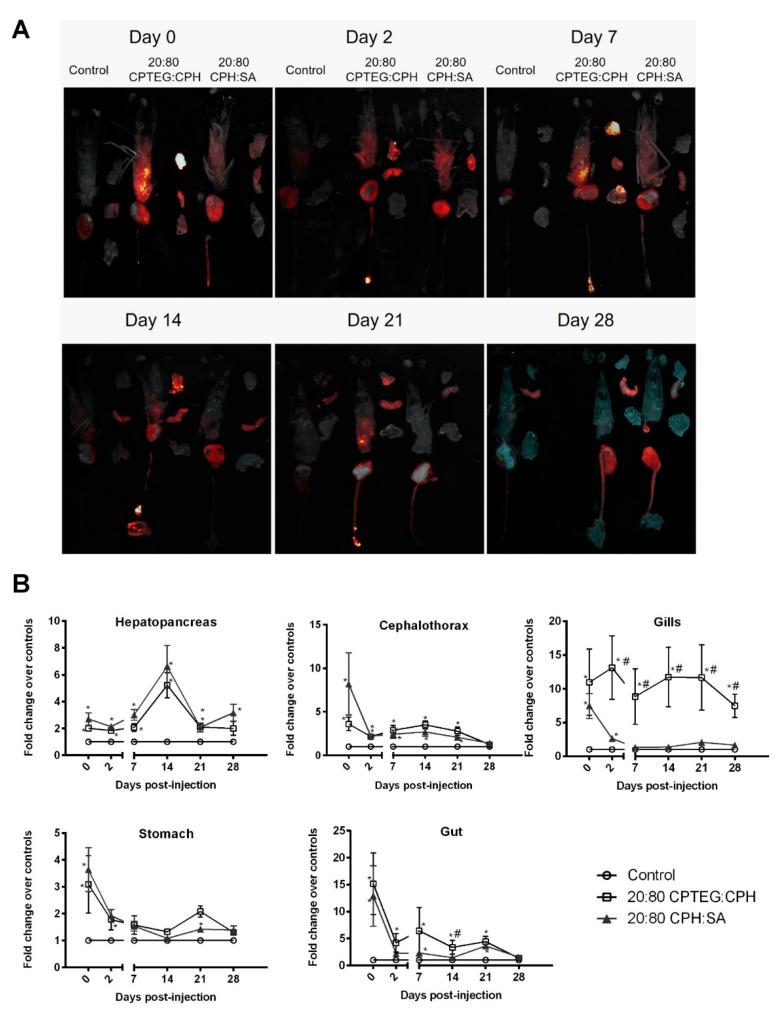
Biodistribution of 1% rhodamine-loaded nanoparticles in *L. vannamei* post-RG administration. Shrimp were injected with 250 μg of either 20:80 CPTEG:CPH or 20:80 CPH:SA rhodamine-loaded nanoparticles by reverse gavage (RG). At indicated time intervals, animals were dissected into cephalothorax, hepatopancreas, gut, gills, and stomach. (**A**) Qualitative image analysis. Tissue samples were placed on an imaging tray and rhodamine fluorescence was captured using Carestream Multispectral Imager. Images shown are representative of results obtained from two independent experiments. (**B**) In vivo fluorescence quantification of rhodamine. Data are expressed as the mean fold change over controls ± SEM of two independent experiments. Statistical differences (*p* < 0.05) of either nanoparticle treatment in comparison to the control are indicated by *, while differences between the two nanoparticle formulations are indicated by #.

Similar to pathogens that can have tissue or organ tropism, the nanoparticles also showed differential spatiotemporal distribution in animals [24]. In order to study the effect of nanoparticle chemistry on their biodistribution in shrimp, animals were injected with 250 µg of 1% rhodamine-loaded 20:80 CPH:SA or 20:80 CPTEG:CPH nanoparticles via reverse gavage. At 2 h following injection, fluorescence from both nanoparticle formulations was observed in the cephalothorax, hepatopancreas, stomach, gut, and gills of the animals (Figure 4A). The highest initial fluorescence in the nanoparticle-injected animals was observed in the gut tissue (~15-fold over controls) followed by the gills (~10 fold over controls).

Although there was reduction in nanoparticle fluorescence in the cephalothorax, stomach, and gut during the first 2 days postinjection, the fluorescence values stabilized between days 2 and 21 (Figure 4B). A nanoparticle chemistry-dependent effect was observed in the gills where animals injected with both nanoparticle formulations having similar fluorescence values at day 0; however, animals receiving the 20:80 CPH:SA nanoparticles demonstrated a reduction in fluorescence to baseline values within a week. In contrast, shrimp injected with the 20:80 CPTEG:CPH nanoparticles continued to exhibit fluorescence even at the termination of the study at day 28 (Figure 4B).

### 3.5. Double-Stranded RNA-Based Nanovaccines Provide Protection against WSSV

**Figure 5:**
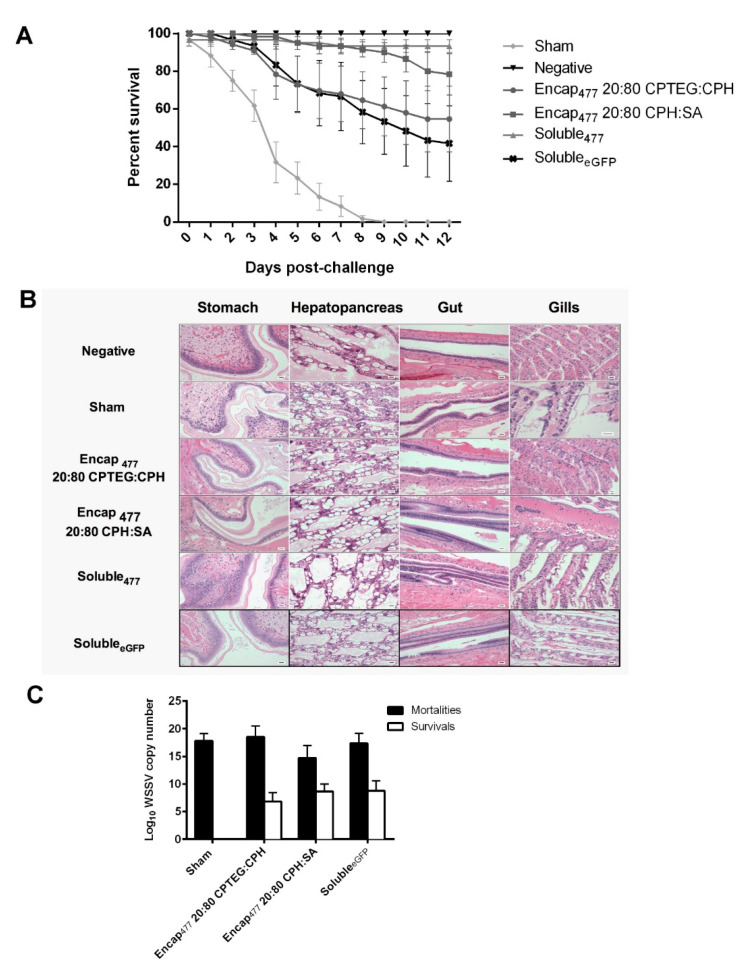
Shrimp survival against WSSV challenge. (**A**) Shrimp were injected by reverse gavage (RG) with 11 μg of either soluble WSSV 477 dsRNA or 11 μg WSSV 477 dsRNA encapsulated in 100 μg 20:80 CPTEG:CPH or 20:80 CPH:SA nanoparticles. Three days postvaccination animals were challenged with a lethal dose of WSSV and survival was monitored through day 12. Sham controls were injected with water prior to challenge. Soluble _eGFP_ was used as non-shrimp specific vaccination control. The negative controls did not receive any viral dose. n = 60 (two independent experiments with 30 animals/treatment) except the negative control group (n = 40). (**B**) Photomicrographs of shrimp tissue postchallenge. Scale bar = 20 μm. (**C**) Viral load measured using WSSV-specific qRT-PCR.

To examine the efficacy of dsRNA-based nanovaccines against WSSV infection in *L. vannamei*, 20:80 CPH:SA and 20:80 CPTEG:CPH nanovaccine formulations were prepared and their performance compared to that of soluble dsRNA, which previously showed high-level protection (96% survivability) against WSSV at 10 days postinjection (DPI) [16]. In the current studies, shrimp that received soluble dsRNA demonstrated 93% survivability, while animals that received the dsRNA-based 20:80 CPH:SA nanovaccine were similarly protected against WSSV with 80% mean survival and animals that received the dsRNA-based 20:80 CPTEG:CPH nanovaccine showed 55% mean survival 12 days postinfection, as shown in Figure 5A. In contrast, there were no survivors in sham control groups at 12 days postinfection. In addition, the administration of the soluble _eGFP_ formulation provided 40% protection against WSSV consistent with previous reports in which heterologous dsRNA provides protective effects against WSSV [34,35]. The mean survival of all vaccinated groups (Encap_477_ 20:80 CPTEG:CPH, Encap_477_ 20:80 CPH:SA and soluble_477_) were significantly different from the sham control group as per Bayesian analysis. At 5% level the credible intervals compared to the sham control are 5.01:642.83 for CPH: SA, 5.43:643.55 for CPTEG:CPH and 6.88:644.40 for Soluble_477_ (Table 4). All negative control animals survived during the full course of these experiments.

In addition, histopathology analysis of stomach, hepatopancreas, gut, and gill tissue revealed severe lesions characteristic of WSSV infection as well as advanced tissue destruction. In contrast, tissues from nanoparticle-vaccinated groups had normal tissue architecture similar to negative controls (Figure 5B and Table 5). Quantitative real-time PCR results showed that the RNA-based nanovaccines reduced the viral load in the challenged animals. Analysis of shrimp mortality showed that the virus actively multiplied inside the animals, which is evident from the increase in viral copy numbers up to 1.6 × 10^8^ WSSV copy numbers/μL from the starting inoculum of ~9.1 × 10^4^ WSSV copy numbers per shrimp. The comparison between shrimp mortality and survival within the vaccinated groups showed that both animals receiving both nanoparticle formulations had significantly (with *p* values lower than 0.02) reduced virus multiplication (Figure 5C).

### 3.6. Informatics Analysis

**Figure 6:**
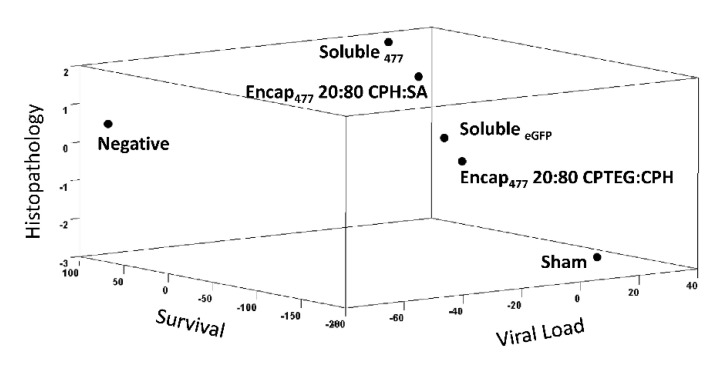
Principal component analysis of viral load, survival, and histopathology. Principal component analysis was used to determine the effects of nanovaccines on key vaccine efficacy parameters: survival, viral load, and histopathology postchallenge. The results were plotted three dimensionally and the similarity among formulations is defined through the comparison of Euclidean distances, where formulations in close proximity to one another represent greater similarity.

Principal component analysis was employed to analyze the concurrent effects of nanovaccines on key vaccine efficacy parameters: survival, viral load and histopathology. A plot incorporating data from the WSSV challenge (survival), qPCR analysis (viral load) and histopathology on three axes was generated with equal importance given to each axis. Through PCA, the proximity of data points indicated the degree of correlation among formulations. As expected, the negative control and sham vaccinated groups were identified as having very different characteristics, demonstrated by the largest distance between these two data points (Figure 6 and Figure S1). The negative control group was highly correlated with Encap_477_ 20:80 CPH:SA and Soluble_477_ on the histopathology and survival axes. The ranking of the highest to lowest similarity compared to unchallenged negative control group was: Encap_477_ 20:80 CPH:SA; Soluble_477_; Soluble_eGFP_; Encap_477_ 20:80 CPTEG:CPH; and sham (Figure 6).

## 4. Discussion

Even though nanomedicine has already made notable contributions to human health, its use in promoting animal health is still in its infancy. Numerous studies have shown that polyanhydride nanoparticles are excellent vehicles for antigen delivery by providing antigen stability, controlled release, intrinsic adjuvanticity, and protective immunity [36,37]. However, this is the first study to our knowledge demonstrating their use as dsRNA delivery vehicles for prophylactic vaccination strategies in invertebrates.

Polyanhydride nanoparticles were shown to be suitable for the encapsulation and release of dsRNA. While both the 20:80 CPH:SA and 20:80 CPTEG:CPH nanoformulations provided near-zero order dsRNA release kinetics (Figure 1), the encapsulation efficiency of dsRNA was much greater in the 20:80 CPTEG:CPH nanoparticles. CPTEG-containing polymers are more amphiphilic (in comparison with the hydrophobic CPH:SA copolymers), and thus, more compatible with protein antigens [32,38]. It is possible that dsRNA, with a hydrophilic backbone, may have higher thermodynamic compatibility with the amphiphilic CPTEG moieties leading to a higher encapsulation efficiency than that observed in 20:80 CPH:SA particles.

One of the foremost concerns for feed animal vaccines is that they should not cause any adverse effects to the final yield. Our data demonstrate that neither of the two nanoformulations tested induced any untoward effects in shrimp injected via reverse gavage, even with a dose of nanoparticles that was five-fold higher than that required for vaccine delivery. Blinded histopathological analysis confirmed that no histological abnormalities were caused in the shrimp alimentary tract tissue or gills due to nanoparticles. Hemocytic congestion, a marker of inflammation in shrimp, was also evaluated and found to be no different than that in control animals (Figure 3B and Table 3). Finally, shrimp weight gain, which is the most important contributor to the farm yield, showed that particles did not affect the normal shrimp development (Table 2). These results are in accordance with previous work demonstrating safety of polyanhydride nanoparticles in a murine model [39].

The effect of chemistry on nanoparticle uptake and distribution in mammalian systems has been extensively evaluated [17,39]. Like a pathogen that can have tissue or organ tropism, polyanhydride particles can also show differential spatiotemporal distribution [17,40]. We tested if the nanoparticle chemistry impacts tissue biodistribution and persistence in *L. vannamei*. As expected, nanoparticles were present in the gut, no doubt facilitated by the route of administration; however, within 2 h they were also visualized in the stomach, hepatopancreas, and cephalothorax. At day 0, depending on the tissue, both CPH:SA and CPTEG:CPH particles exhibited fluorescence that was 2–15 fold higher compared to the controls and no significant differences were observed between the two chemistries. Interestingly, at day 0, gill tissues showed very high fluorescence levels with both 20:80 CPH:SA and 20:80 CPTEG:CPH nanoparticles. While our previous work has demonstrated that the particles migrate systemically [39] in other animal models, the mechanism(s) by which the nanoparticles trafficked to the gills remains to be determined. It is important to note that gills are one of the target organs for several viral pathogens such as Taura syndrome virus (TVS), yellowhead virus (YHV) and WSSV [4,41]. Thus, the presence of nanoparticles in gills potentially contributes to protection against diseases caused by these viruses.

Nanoparticle persistence is another important outcome to enable vaccinated animals to be protected from the disease through different life stages (post-larva, juvenile, or adult). Our previous work has shown that polymer properties such as glass transition temperature (T_g_), and hydrophobicity can significantly affect the tissue residence time of nanoparticles in mice [39]. Both CPH:SA and CPTEG:CPH nanoparticles persisted in alimentary tract tissue and gills for at least 21 days postinjection. The combination of long persistence of particles along with short life span of shrimp may compensate for the lack of adaptive immunological memory and provide protection during the grow-out phase of farming. The reduction in fluorescence observed between day 0 and day 2 may likely be due to excretion of particles and the initial burst release of rhodamine from the particles. A distinct nanoformulation chemistry effect was observed in the gills where 20:80 CPH:SA particle fluorescence returned to baseline levels by day 7 postinjection, while the CPTEG:CPH particles showed high intensity even 28 days later.

Vaccines based on dsRNA can be effectively used against several shrimp viruses [5], however development of suitable delivery strategies for mass vaccination of shrimp in aquatic systems is a major challenge. Oral administration to shrimp (i.e., through feed) is by far the most appealing method of vaccine delivery. These experiments demonstrated that our nanovaccine when administered directly to the gut provided significant protection against a lethal dose of WSSV. A reverse gavage strategy for administration was used to mimic oral delivery and the results indicated that the nanovaccines protected encapsulated dsRNA from the gut environment and delivered stable and functional WSSV RNAi trigger. The nanovaccines not only increased the survivability of challenged shrimp but also reduced WSSV replication in vivo. This finding can have important implications in the field as it is known that horizontal transmission of WSSV in a shrimp pond can either be by direct contact of infected cadaver i.e., by cannibalism or by indirect water-borne route [42]. Regardless of the route of transmission, the reduced virus load may thus contribute to abating the spread of WSSV in field conditions by lowering the available viral dose.

Although the 20:80 CPH:SA nanovaccine provided greater protection against infection in this work than the 20:80 CPTEG:CPH nanovaccine, it is important to understand the advantage of each nanoformulation. For example, the dsRNA release kinetics from the 20:80 CPH:SA nanoparticles resulted in a large initial burst, such that almost 90% of the encapsulated dsRNA was released rapidly (Figure 1). Due to this large burst, high amounts of dsRNA were likely available in the shrimp alimentary tract at the time of infection (72 h post-vaccination) and enhanced protection. In contrast, the initial burst of dsRNA from the 20:80 CPTEG:CPH nanovaccine was lower in comparison to that from the 20:80 CPH:SA nanovaccine. The CPH-rich chemistries are known to have slower erosion rates due to their inherent hydrophobicity, and thus, reduced release rates of encapsulated payloads [32,33]. As demonstrated by the dsRNA release kinetics (Figure 1) and persistence of polyanhydride nanoparticles (Figure 4), we postulate that 20:80 CPTEG:CPH nanovaccine would sustain the release of dsRNA (and thus availability of dsRNA within the shrimp) and enhance protection against infection at later time points (i.e., several months) in comparison with 20:80 CPH:SA nanoparticles. In contrast, the early infection schedule (i.e., 72 h post vaccination) is advantageous to the 20:80 CPH:SA nanovaccine. In order to design a single nanoformulation that can provide protective immunity against both early and late infections, it is conceivable that administering a cocktail of both the 20:80 CPH:SA and 20:80 CPTEG:CPH nanoformulations will enable both early and long-lasting immunity.

Despite the reduced encapsulation efficiency, the 20:80 CPH:SA nanovaccine provided protection against viral challenge similar to that provided by the soluble_477_ RNA (~80%) demonstrating the dose sparing capabilities of the nanovaccine. This result is consistent with previous studies in our laboratories with 20:80 CPH:SA microparticles, in which we observed a 64-fold antigenic dose sparing capability in mice, compared to antigen alone [11]. The encapsulation efficiency data (Table 1) indicate that shrimp administered with 20:80 CPH:SA nanovaccine received approximately 4 µg of dsRNA, compared to shrimp that received soluble_477_ dsRNA alone (11 µg). However, upon infection with WSSV, both 20:80 CPH:SA and soluble_477_ dsRNA treated animals had similar rates of survival (Figure 5). Therefore, 20:80 CPH:SA nanovaccine provided similar levels of disease protection with third the amount of dsRNA. Furthermore, an important ramification of these data is the use of the nanovaccine in the field settings where it can be mixed with shrimp feed. Previous data from our lab have shown that polyanhydride nanovaccines can protect the encapsulated cargo and be efficacious even when stored at relatively high temperature of 40 °C for at least 4 months [36]. Since shrimp farming is done in warmer temperatures (~30 °C), the temperature stability of the nanovaccine can be instrumental for application in the temperate/tropical climate. In contrast, dsRNA exposed to ultraviolet light loses its biological activity and structure in a matter of few hours [43].

Altogether, these data demonstrate the ability of the polyanhydride nanoparticle delivery platform to deliver dsRNA antivirals to aquatic animals. WSSV, being the most threatening of the shrimp viruses, was used in this study, however, the rapid tailoring of dsRNA against other viruses can make this adaptable to other diseases too. Moreover, the versatility offered by this technology to deliver a wide range of antibiotic, antiviral, and antifungal molecules have the potential of improving global agriculture by disease control in crustacean and finned fisheries settings. The end result is a novel product that ultimately provides more effective, lower-dose therapies to benefit animal health, with tremendous potential to thereby contribute to both enhanced food safety and security and environmental health.

## Figures and Tables

**Table 1:** Polyanhydride Nanoparticle Characterization.

Chemistry	Structure	Scanning Electron Microscopy	Diameter (Mean ± SEM)	Encapsulation Efficiency (Mean ± SEM)
20:80 CPH:SA + 1% Rhodamine	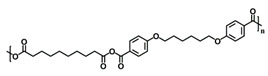	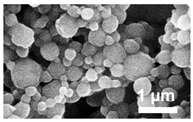	273 ± 126 nm	N/A
20:80 CPTEG:CPH + 1% Rhodamine	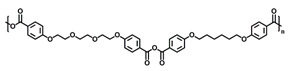	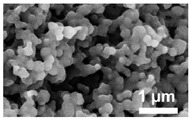	219 ± 74 nm	N/A
20:80 CPH:SA + 11% dsRNA	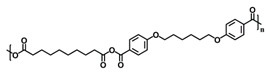	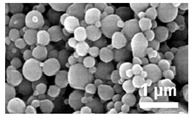	290 ± 160 nm	33.8 ± 1.2%
20:80 CPTEG:CPH + 11% dsRNA	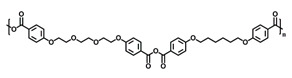	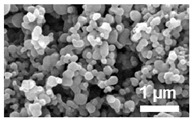	164 ± 66 nm	74.0 ± 1.0%

**Table 2:** Shrimp weight gain post-nanoparticle administration.

Group	Average Initial Weight (W_I_) (gm)	Average Final Weight (W_F_) (gm)	Weight Gain (W_F_ − W_I_) (gm)	% Weight Gain [(W_F_ − W_I_)/W_I_] × 100
Control	5.01 ± 0.18	9.10± 0.34 (a)	4.08	81.4
20:80 CPTEG:CPH	4.80 ± 0.16	10.52 ± 0.21 (b)	5.73	119.4
20:80 CPH:SA	4.99 ± 0.17	10.17 ± 0.35 (b)	5.18	103.76

Data are represented as mean ± SEM. Treatments with different letters are significantly different from one another at *p* < 0.05.

**Table 3:** Summary of histological findings in shrimp administered with 500 μg of blank nanoparticles.

Parameter	Control	20:80 CPTEG:CPH	20:80 CPH:SA
Hepatopancreas vacuolization	0.62 ± 0.18	0.85 ± 0.14	1.00 ± 0
Enteric bacteria	1.25 ± 0.47	1.33 ± 0.66	1.50 ± 0.28
Septic hepatopancreatic necrosis	0	0	0.50 ± 0.50
Hemocytic nodules	0.25 ± 0.16	0.42 ± 0.20	0.37 ± 0.26
Hemocytic congestion	0.12 ± 0.12	0.28 ±0.18	0.37 ± 0.18
Skeletal muscle necrosis	0.75 ± 0.31	0.14 ± 0.14	0.87 ± 0.29
Lymphoid organ spheroids	2.00 ± 0.0	1.75 ± 0.25	0.75 ± 0.25

Data are represented as mean ± SEM.

**Table 4:** The pairwise treatment differences from Bayesian analysis.

Treatment 1	Treatment 2	Estimate	Standard Deviation	Lower HPD	Upper HPD
Encap_477_ 20:80 CPTEG:CPH	Sham	251.84	192.17	5.0118	642.83
Encap_477_ 20:80 CPH: SA	Sham	252.48	192.18	5.4316	643.55
Soluble_477_	Sham	253.66	192.16	6.8842	644.4
Negative	Sham	930.27	586.33	97.4592	2135.09

**Table 5:** Severity grade of necrosis and lesions caused by WSSV postchallenge.

Group	Hepatopancreas Vacuolization	Lymphoid Organ Spheroids	Hemocytic Nodules	Skeletal Muscle Necrosis	WSSV Lesions
Negative	1	0.5 ± 0.5	0	0	0
Sham	1.6 ± 0.33	0	0	0.66 ± 0.66	2.66 ± 1.33
Encap_477_ 20:80 CPTEG:CPH	1.6 ± 0.33	1 ± 0.57	0.33 ± 0.33	0	0
Encap_477_ 20:80 CPH:SA	0	0	1 ± 1	0	0
Soluble_477_	1	0.66 ± 0.66	1 ± 1	0	0
Soluble_eGFP_	1.6 ± 0.33	2 ± 0.57	0	0	0

Data represented as mean ± SEM.

## Data Availability

All data are available on request. Please contact: lyric.bartholomay@wisc.edu.

## Supplementary Materials

The following supporting information can be downloaded at: https://www.mdpi.com/article/10.3390/vaccines10091428/s1, Figure S1 Results of principal component analysis, with the similarity of systems shown through scores and the relevance of the descriptors in differentiating the impact of changing conditions defined through loadings.
