# Supplementary material for: RNA Nanovaccine Protects against White Spot Syndrome Virus in Shrimp
Source: Vaccines (Basel). 2022 Aug 30;10(9):1428. doi: 10.3390/vaccines10091428 (PMC9504209; doi:10.3390/vaccines10091428)
Supplement: Supplementary file 1 [file vaccines-10-01428-s001.zip › vaccines-1821061-supplementary.pdf]

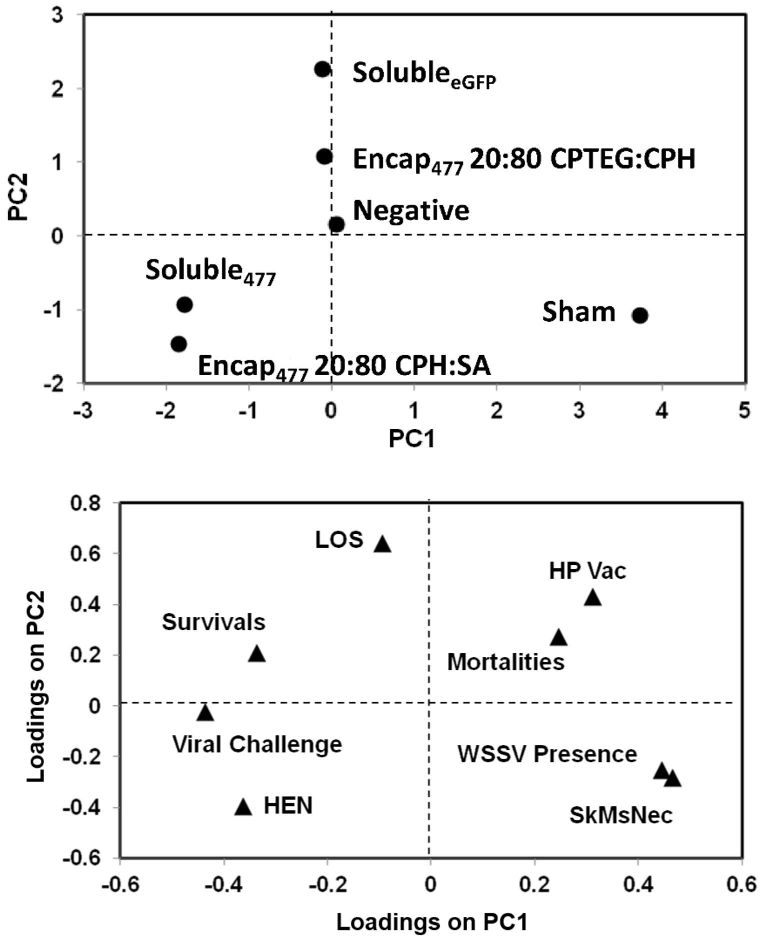

Figure S1. Principal component analysis on unified data set of viral load, survival, and istopathology. Figure 6 provided separate analyses on each category of data in order to assess their separate roles. In this figure, the categories have been analyzed together, with the correlations still defined through Euclidean distance for the scores plot (Top), while correlation in the loadings plot (Bottom) are defined by the cosine of the angle between any two points and the origin. This suggests CPH:SA decreases mortality values the most (ie. increasing mortalities contributes to increasing PC1 and PC2 values).
